# Mitochondrial metabolic reprogramming in colorectal cancer: mechanisms of resistance and future clinical interventions

**DOI:** 10.1038/s41420-025-02670-y

**Published:** 2025-08-09

**Authors:** Xiuxiu Qiu, Ao Wang, Jiahui Wang, Zhanxia Zhang, Li Tao

**Affiliations:** 1 Department of Oncology, Longhua Hospital, Shanghai University of Traditional Chinese Medicine, Shanghai, China; 2 Department of Nephrology, Yueyang Integrated Traditional Chinese and Western Medicine Hospital Affiliated to Shanghai University of Traditional Chinese Medicine, Shanghai, China; 3 Cancer Institute, Longhua Hospital, Shanghai University of Traditional Chinese Medicine, Shanghai, China

**Keywords:** Gastrointestinal cancer, Colorectal cancer

## Abstract

Colorectal cancer (CRC) is a leading cause of global cancer mortality, with therapeutic resistance constituting a major barrier to sustained clinical benefit. Mitochondrial metabolic reprogramming has emerged as a central adaptive mechanism that enables CRC cells to withstand hypoxia and therapeutic pressure, while concurrently driving resistance to chemotherapy, targeted agents, and immunotherapy. In this Review, we explore how mitochondrial metabolism contributes to therapeutic resistance, with particular emphasis on metabolic plasticity, redox balance, and organelle quality control. We also assess enabling technologies such as spatial transcriptomics, proteomics, and patient-derived organoids, and discuss their translational relevance in stratifying metabolic vulnerabilities and informing individualized therapies. Targeting mitochondrial rewiring represents a compelling strategy to overcome resistance and drive progress toward personalized CRC therapy.

## Facts

- Mitochondrial metabolic reprogramming drives resistance to chemotherapy, targeted therapy, and immunotherapy in CRC, partly via oncogenic pathways like KRAS–MAPK.
- Mitochondria support CRC adaptation and metastasis through metabolic plasticity, redox homeostasis, organelle quality control, and microRNA-mediated post-transcriptional regulation.
- Technologies such as spatial transcriptomics, proteogenomics, PDOs, and AI-based multi-omics are enhancing the understanding and targeting of mitochondrial metabolism in CRC.
- Targeting mitochondrial adaptations is considered a promising avenue for overcoming resistance and advancing personalized therapeutic strategies in CRC treatment.

## Open questions

- What are the specific molecular regulators that govern mitochondrial metabolic plasticity under therapeutic stress in CRC?
- How can we systematically decode the dynamic impact of mitochondrial metabolic heterogeneity on therapeutic resistance and individualized intervention outcomes in CRC?
- What combinatorial strategies (e.g., mitochondrial inhibitors plus immunotherapy) show the most promise in preclinical or early clinical studies for CRC?
- In the future clinical translation of mitochondrial metabolism-targeted therapies, what are the major technological bottlenecks and biological unknowns that need to be addressed?

## Introduction

Colorectal cancer (CRC) ranks as the third most commonly diagnosed malignancy worldwide, with approximately 1.93 million new cases and 904,000 deaths in 2022 [1]. By 2040, the global burden is projected to exceed 3.2 million new cases and 1.6 million deaths annually [2]. Despite advances in surgical techniques, chemoradiotherapy, targeted agents, and immunotherapies that have improved outcomes for some patients, intratumoural heterogeneity and adaptive resistance continue to limit durable responses in many cases [3]. For instance, activating mutations in KRAS or BRAF markedly reduce the efficacy of EGFR inhibitors, illustrating the limitations of current approaches in addressing the molecular complexity of CRC [4].

In recent years, metabolic reprogramming has been recognized as a fundamental strategy by which cancer cells sustain rapid proliferation and adapt to hostile microenvironmental conditions. While the classical Warburg effect highlights glycolysis as the primary metabolic pathway in cancer, a growing body of evidence demonstrates that mitochondrial function is equally indispensable, especially under therapeutic stress and in metastatic contexts. In CRC, the reactivation or remodeling of mitochondrial metabolic pathways, particularly oxidative phosphorylation (OXPHOS) and fatty acid oxidation (FAO), is closely associated with key oncogenic processes such as the evasion of apoptosis, the maintenance of cancer stem cell properties, and the regulation of redox homeostasis [5–8]. These metabolic adaptations provide a foundation for the development of therapeutic resistance.

Further studies have identified microRNAs (miRNAs) as key post-transcriptional regulators of mitochondrial metabolism in CRC [9]. By targeting key enzymes involved in OXPHOS, FAO, and redox homeostasis, miRNAs can alter mitochondrial function and reprogram cellular metabolism [10–12]. These metabolic changes not only disrupt energy production but also enhance therapeutic resistance and metastatic potential by modulating reactive oxygen species (ROS) levels and apoptosis signaling pathways.

Moreover, CRC cells exhibit marked metabolic plasticity, in which FAO serves as a key mitochondrial mechanism for energy compensation [13, 14]. FAO not only supports ATP production but also promotes tumor progression, metastasis, and therapeutic resistance by regulating ROS levels, lipid metabolism-related transcription factors such as peroxisome proliferator-activated receptors (PPARs), and components of the AMP-activated protein kinase (AMPK) signaling axis [15, 16]. Therefore, the regulation of FAO-related enzymes by miRNAs may further exacerbate therapeutic resistance and metastatic potential in CRC.

Notably, the spatiotemporal metabolic heterogeneity of CRC has attracted increasing attention. Emerging evidence indicates marked differences between primary and metastatic lesions in terms of metabolite composition, mitochondrial activity, ROS levels, and the architecture of the tumor microenvironment [17–20]. These dynamic metabolic variations directly influence drug distribution, target expression, and therapeutic response, posing significant challenges to the development of effective personalized treatment strategies [15, 18, 21].

A deeper understanding of mitochondrial metabolic reprogramming in CRC, particularly its involvement in therapeutic resistance, metastatic adaptation, and metabolic vulnerabilities [14], is essential for the development of targeted interventions and the advancement of precision oncology.

Although numerous previous reviews have explored metabolic reprogramming in cancer, most have predominantly focused on glycolysis or broad metabolic alterations common across multiple tumor types. For instance, Faubert et al. provided a comprehensive overview of cancer metabolism, emphasizing glycolytic and glutamine-dependent pathways that support tumor progression [22]. Zhang et al. specifically addressed metabolic reprogramming in CRC, yet their analysis encompassed a wide range of pathways without a focused examination of mitochondrial metabolism or its role in therapy resistance [23]. The importance of mitochondrial pathways was further emphasized in a pan-cancer review by Arsenian-Henriksson and colleagues, who outlined the central role of mitochondrial metabolism in tumor biology and highlighted diverse therapeutic opportunities [24]. However, this analysis did not specifically address CRC-associated metabolic adaptations or resistance mechanisms. More recently, Wang et al. investigated the pharmacological modulation of mitochondrial function in intestinal diseases, including CRC, offering insight into mitochondrial dynamics and bioenergetics [25]. However, their discussion gave limited attention to how mitochondrial rewiring intersects with oncogenic drivers such as KRAS or BRAF, or how it facilitates immune evasion and metastasis.

While recent studies underscore a growing focus on mitochondrial metabolism in oncology, they also reveal a critical gap: comprehensive analyses addressing mitochondrial metabolic rewiring in CRC and its mechanistic links to therapeutic resistance and clinical translation remain limited.

To address this gap, this review explores four key areas: (1) the core mechanisms of mitochondrial metabolic reprogramming in CRC and its role in driving therapeutic resistance; (2) the functional networks of energy metabolism, redox balance, and organelle quality control in modulating treatment response; (3) emerging intervention strategies targeting mitochondrial metabolism, including small-molecule inhibitors, combination therapies, and translational opportunities; and (4) the integration of advanced technologies, including AI-assisted diagnostics, multi-omics approaches such as spatial transcriptomics and proteomics, and patient-derived organoids (PDOs), is driving metabolism-guided precision medicine. Collectively, these perspectives aim to provide a timely, CRC-specific, and mechanism-based framework for developing clinically actionable strategies targeting mitochondrial metabolism.

## Core mechanisms of mitochondrial metabolic reprogramming

### Supplying flexible fuel: metabolic plasticity and therapeutic resistance

CRC cells exhibit pronounced metabolic plasticity, enabling dynamic switching between glycolysis and mitochondrial OXPHOS in response to fluctuations in oxygen, glucose, and other metabolic substrates, thereby adopting a hybrid metabolic phenotype [6]. This metabolic plasticity not only enhances the survival of cancer cells under extreme conditions such as hypoxia and nutrient deprivation but also provides a critical foundation for therapeutic evasion and the acquisition of drug-resistant phenotypes [13].

Studies have demonstrated that the coordinated activation of the ROS/PI3K/Akt and Wnt/β-catenin signaling pathways can induce the expression of HIF-1α, driving glycolysis-centered metabolic reprogramming. This enhances cancer adaptability and survival advantage under 5-fluorouracil (5-FU) treatment [26]. Moreover, cancer stem cells (CSCs) tend to rely on OXPHOS rather than glycolysis as their primary energy source, a metabolic trait that confers enhanced therapeutic tolerance and tumor-initiating capacity [27].

Therapy-induced metabolic reprogramming also plays a central role in the induction of cellular dormancy, epithelial–mesenchymal transition (EMT), and the emergence of drug-tolerant persister cell populations [28]. These persister cells typically exhibit low metabolic activity and reduced sensitivity to therapy, yet they possess a remarkable capacity for survival. They can be reactivated following treatment withdrawal, often contributing to tumor relapse [29]. At the molecular level, AMPK plays a pivotal role in the metabolic stress response. By sensing changes in the intracellular ATP/AMP ratio, AMPK activates FAO and mitochondrial respiration, enhancing cellular adaptation to energy deprivation [30, 31]. In contrast, the PI3K–AKT–mTOR pathway, a prototypical pro-proliferative signaling axis, primarily promotes glycolysis and anabolic biosynthesis [32]. These processes are dynamically balanced with catabolic pathways, collectively conferring metabolic flexibility that is essential for the survival and growth of cancer cells [32].

Multiple preclinical studies have demonstrated that co-targeting glycolysis and mitochondrial metabolism, or modulating key metabolic regulators such as AMPK, can significantly enhance the sensitivity of CRC cells to chemotherapy and targeted agents. This approach could delay tumor recurrence and progression [23]. Therefore, the plasticity of energy metabolism is not only a hallmark of CRC cell adaptability but also a fundamental driver of therapeutic resistance [19]. Elucidating the metabolic regulatory networks will offer a foundational framework and actionable targets for the development of next-generation metabolism-targeted therapies.

### Defending the balance: redox homeostasis under therapeutic stress

The maintenance of redox homeostasis under metabolic and oxidative stress conditions is essential for cancer cell survival [33]. In CRC, ROS levels are frequently elevated due to enhanced mitochondrial activity, activation of oncogenic signaling pathways, and chronic inflammation [34]. Although high levels of ROS can induce DNA damage, lipid peroxidation, and protein dysfunction, low to moderate levels have been shown to act as key signaling molecules that promote cancer cell proliferation, stemness maintenance, and migratory capacity [35].

To counteract therapy-induced oxidative stress, CRC cells activate multiple antioxidant defense networks, among which the Keap1–Nrf2 pathway plays a central role [36]. Under oxidative conditions, Nrf2 escapes Keap1-mediated ubiquitination and translocates into the nucleus, where it induces the expression of antioxidant genes involved in glutathione (GSH) synthesis, NADPH production, and cellular detoxification [34].

This pathway is frequently hijacked in CRC, serving as a major protective mechanism against the cytotoxicity of ROS-inducing chemotherapeutic agents such as 5-FU and oxaliplatin. In addition, other antioxidant systems—including GSH, thioredoxin (Trx), and peroxiredoxins—are highly active in CRC cells and collectively reinforce their defense against oxidative stress [34, 37, 38].

Importantly, the regulation of redox homeostasis is tightly coupled with metabolic reprogramming [23]. Under conditions of hypoxia, nutrient deprivation, or impaired mitochondrial function, cancer cells modulate NADPH and GSH levels through key metabolic pathways such as the pentose phosphate pathway (PPP), FAO, and glutamine metabolism, thereby establishing a metabolic–redox feedback loop [39–41]. This adaptive circuitry enables cancer cells to continuously remodel their metabolic architecture under therapeutic pressure, maintaining ROS balance and promoting survival [34].

From a therapeutic perspective, disrupting redox regulatory mechanisms in CRC cells is emerging as a promising strategy to overcome treatment resistance [42]. Inhibition of Nrf2 activity, depletion of intracellular GSH reserves, or induction of lipid peroxidation-associated ferroptosis have shown potential in preclinical studies to enhance chemosensitivity and reverse resistance [43, 44]. Thus, redox regulation represents not only a key survival mechanism for cancer cells, but also a potential metabolic vulnerability [45]. Targeting antioxidant pathways may therefore offer a novel avenue for CRC treatment in the future.

### Sustaining the engine: mitochondrial quality control and transport networks

As the central “engine” of cellular bioenergetics and intermediary metabolism, mitochondrial functional integrity is critical for sustaining high metabolic flux, anabolic potential, and therapeutic responsiveness in CRC [18]. Recent studies have revealed that dysregulation of mitochondrial dynamics—including impaired mitophagy, imbalances in mitochondrial fusion and fission, and defects in mitochondrial biogenesis—is closely associated with CRC initiation, progression, and resistance to therapy [46].

Mitophagy, in particular, serves as a key mechanism for maintaining metabolic homeostasis by selectively removing damaged mitochondria and limiting the accumulation of ROS [47].In CRC, the PINK1–Parkin-mediated mitophagy pathway and Drp1-driven mitochondrial fission have been shown to act in concert to preserve mitochondrial quality under chemotherapeutic stress [48]. Elevated expression of Drp1 promotes mitochondrial fragmentation, which in turn is strongly associated with mitophagy activation and chemoresistance in CRC cells [46, 49]. Conversely, inhibition of mitophagy can lead to mitochondrial dysfunction, trigger apoptosis, and ultimately increase treatment sensitivity [46].

In addition to quality control, the transport of metabolites between mitochondria and the cytosol plays a critical role in supporting metabolic adaptation in cancer cells [50]. The mitochondrial pyruvate carrier (MPC) regulates the entry of glycolytic pyruvate into the mitochondria, serving as a key node linking glycolysis to OXPHOS [51]. Impaired function of the MPC is frequently observed in specific CRC subtypes, driving a metabolic shift toward glycolysis, enhancing stemness, and conferring resistance to OXPHOS inhibitors [52, 53].

Moreover, transporters located on the mitochondrial inner membrane serve as essential carriers for a variety of metabolic substrates [54]. The SLC25 family encodes 53 such transporters in humans, which are broadly involved in the transmembrane transport of metabolic intermediates such as citrate, malate, and glutamate, which are differentially expressed in CRC subtypes and may influence treatment response [55]. Chen et al. found that SLC25A5 regulates mitochondrial ATP/ADP exchange, maintains mitochondrial functional integrity, and promotes metabolic adaptation and proliferation of CRC cells [56]. This finding highlights the central role of the SLC25 family in the “mass-transport” regulatory network, and provides a novel therapeutic strategy for overcoming drug resistance by targeting mitochondrial transporters [56].

Targeting mitochondrial dynamics and metabolic flux regulation offers a novel strategy to disrupt cancer cell metabolic plasticity and enhance therapeutic efficacy [46]. Preclinical studies have shown that inhibiting mitophagy or restoring MPC function can effectively suppress tumor cell viability and synergize with conventional chemotherapeutics such as 5-FU and oxaliplatin [48, 50]. Thus, interfering with the mitochondrial “quality–transport” network in CRC represents a promising approach to overcome metabolism-driven therapeutic resistance.

**Fig. 1:**
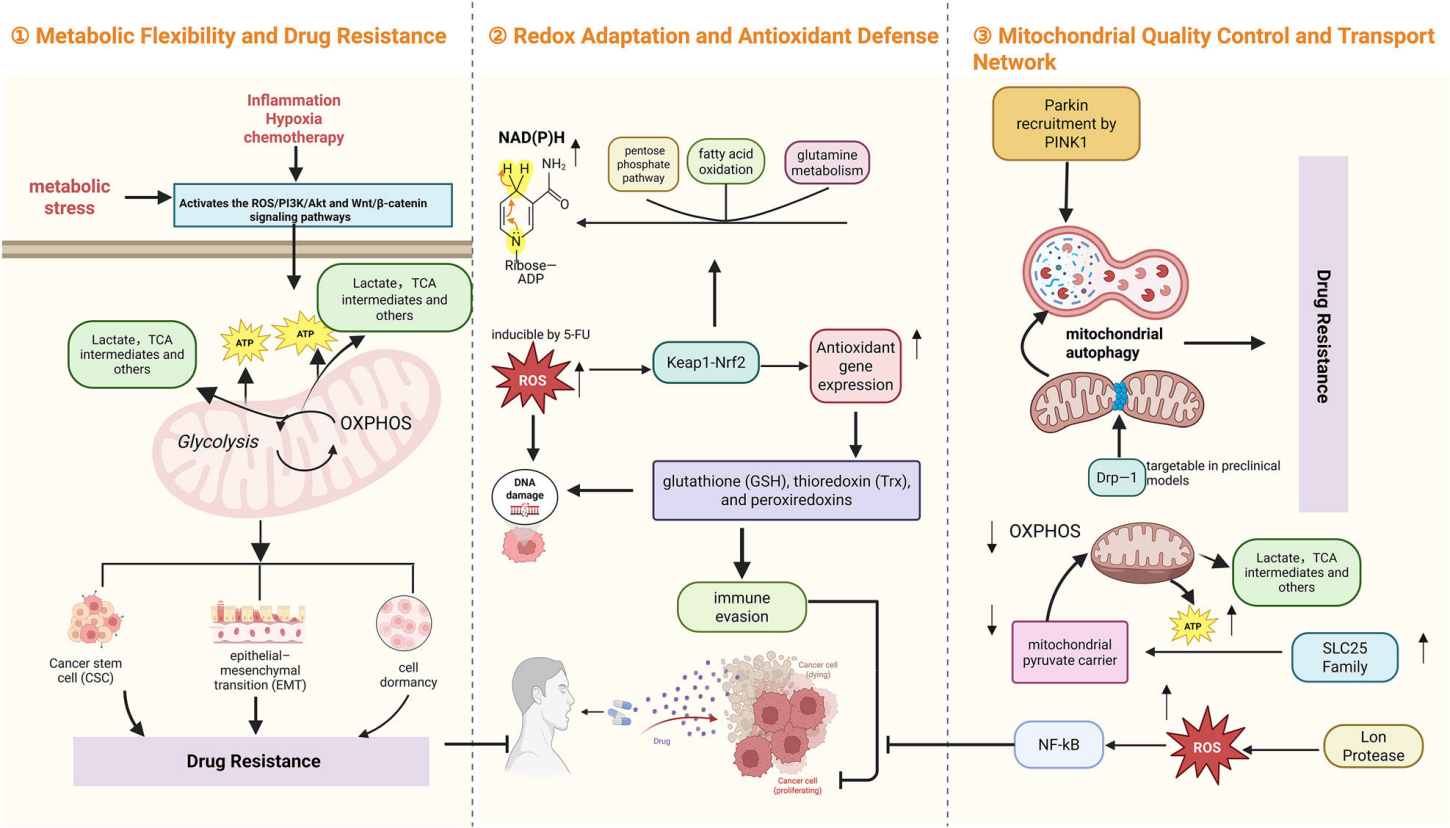
Core mechanisms of mitochondrial metabolic reprogramming. (1) Under metabolic stress (e.g., inflammation, hypoxia, and chemotherapy), CRC cells flexibly switch between glycolysis and OXPHOS to maintain stemness, drive EMT, and support dormancy. (2) Chemotherapy-induced ROS activates the Keap1–Nrf2 pathway, enhancing antioxidant defenses (e.g., GSH, Trx, and peroxiredoxins) to maintain redox balance, promote immune evasion, and confer drug resistance. (3) Mitochondrial quality is preserved through PINK1–Parkin-mediated mitophagy and Drp1-dependent fission, while metabolite transport via MPC and SLC25 family proteins sustains energy homeostasis. Excess ROS can also activate NF-κB via Lon protease, supporting cell survival under therapeutic pressure.

Notably, mitochondrial quality control is not only essential in the context of therapeutic resistance but is also closely implicated in tumor initiation and progression [57]. Recent studies have identified that the mitochondrial AAA protease Lon interacts with multiple protein partners to promote the generation of ROS, which in turn activates the NF-κB signaling axis, amplifying downstream oncogenic pathways and contributing to tumor development and immune evasion [58]. These findings further underscore the central role of mitochondrial quality control in the multistep evolution of CRC (Fig. 1).

## Mitochondrial metabolic reprogramming in CRC: mechanisms of therapy resistance and emerging interventions

**Fig. 2:**
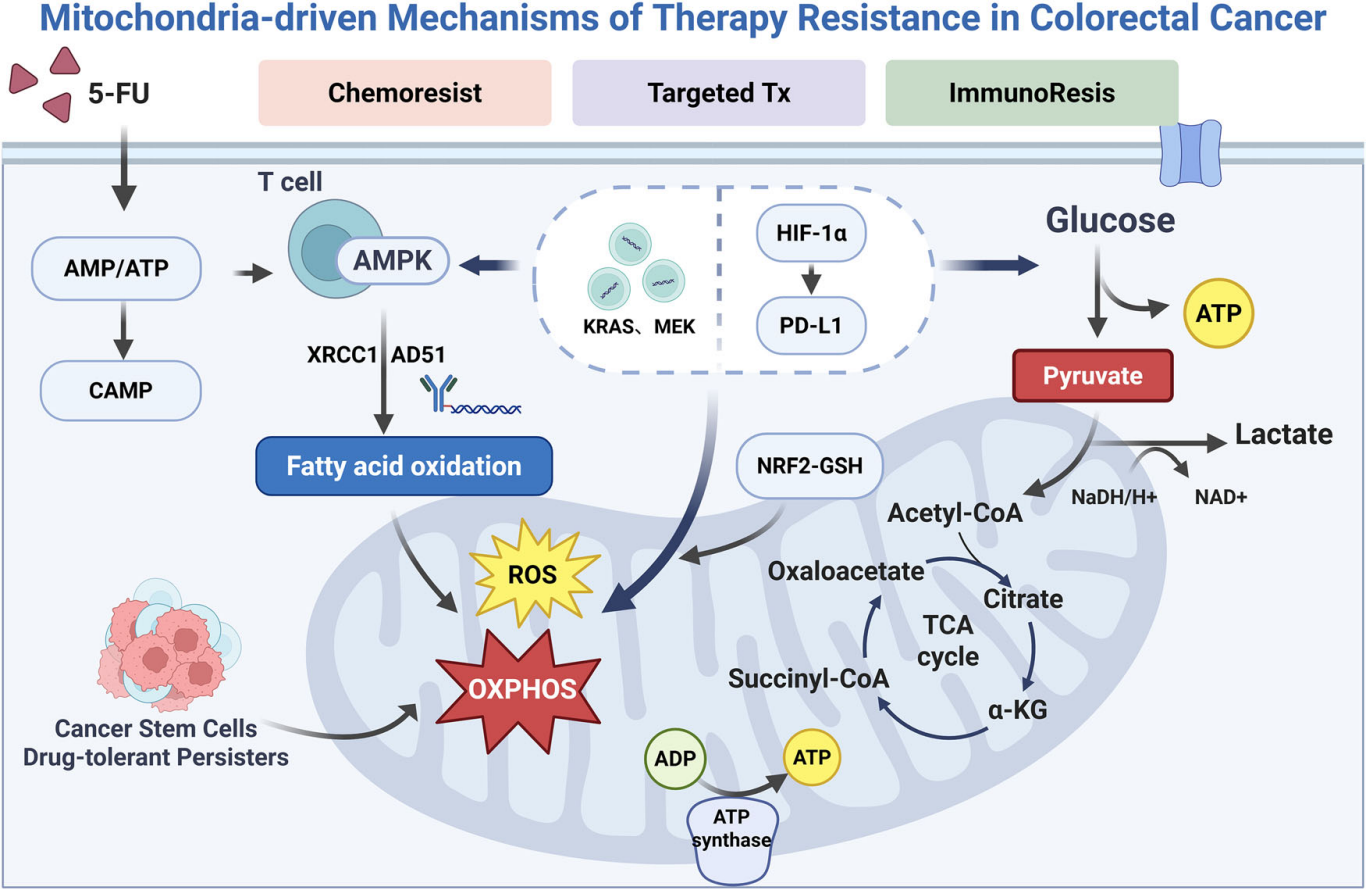
Mitochondria-driven mechanisms of therapy resistance in CRC. CRC cells adapt to chemotherapy, targeted therapy, and immunotherapy by rewiring mitochondrial metabolism. Key processes include AMPK-driven FAO/OXPHOS activation, KRAS/MEK reactivation, HIF-1α–mediated immune evasion, and nutrient depletion in the tumor microenvironment, collectively sustaining therapy resistance.

As discussed above, CRC cells adapt to hypoxia, nutrient deprivation, and therapeutic stress through mitochondrial metabolic reprogramming, which maintains redox homeostasis and organelle quality control to promote survival. This section examines how mitochondrial metabolism contributes to therapeutic resistance across chemotherapy, targeted therapy, and immunotherapy, and considers its potential as a clinically relevant target for intervention (Fig. 2). In addition, we highlight its role in driving metastatic progression, underscoring its dual influence on treatment failure and tumor dissemination.

### Mitochondrial metabolism and KRAS–MAPK axis in CRC chemoresistance

Although chemotherapeutic agents such as 5-FU, oxaliplatin, and irinotecan remain central to CRC treatment, the metabolic adaptability of tumor cells continues to undermine their long-term efficacy [23]. Emerging evidence increasingly highlights the pivotal role of mitochondrial function in driving chemoresistance [48].

CSCs represent a dominant subpopulation within residual tumors following chemotherapy and exhibit a strong reliance on OXPHOS to sustain ATP production and evade apoptosis induced by ROS [59]. The AMPK signaling axis plays a critical role in promoting CSC survival under metabolic stress by stimulating FAO and enhancing mitochondrial respiratory chain activity, thereby maintaining stemness and metabolic adaptability [60]. Disruption of the AMPK–OXPHOS axis has been shown to sensitize CSCs to chemotherapy, underscoring its potential as a therapeutic target [61].

Additionally, a subset of CRC cells can enter a quiescent state under chemotherapeutic stress, giving rise to drug-tolerant persister cells (DTPs) [62]. These cells exhibit high mitochondrial activity and enhanced ROS-buffering capacity, relying on the Trx system and mitophagy to establish a stable metabolic barrier against therapy-induced cell death [63]. Notably, there is functional crosstalk between metabolic reprogramming and DNA damage repair pathways [61]. For example, activation of the MAPK pathway can upregulate DNA repair factors such as XRCC1 and RAD51, thereby enhancing resistance to agents like mitomycin C [64]. This suggests that mitochondrial metabolism not only regulates cellular energy status but also plays a critical role in maintaining genomic stability.

A recent study further demonstrated that WBP1-deficient CRC cells regained sensitivity to 5-FU following the restoration of mitochondrial function [65], reinforcing a causal link between mitochondrial homeostasis and drug responsiveness. Cell subpopulations with high OXPHOS activity consistently exhibit greater drug resistance, suggesting that mitochondrial metabolic heterogeneity may serve as a critical biomarker for resistance stratification and therapeutic classification [66].

In addition to CSCs and DTPs, oncogenic mutations—particularly KRAS mutations—also play a pivotal role in driving mitochondrial metabolic remodeling and promoting chemoresistance [67–69]. Approximately 40% of CRC patients harbor KRAS mutations, which activate the RAS–RAF–MEK–ERK signaling cascade [67]. This pathway not only promotes proliferation but also alters mitochondrial bioenergetics in ways that support survival under therapeutic stress [70, 71]. Moreover, KRAS mutations can upregulate the expression of mitochondrial quality control proteins and metabolic enzymes via the MAPK pathway, contributing to the maintenance of mitochondrial homeostasis [24, 72]. In male patients with KRAS-mutant CRC, decreased ferritin activity has also been observed, suggesting that these tumors may depend on mitochondrial redox-buffering mechanisms to evade iron-dependent cytotoxic responses [5, 72, 73].

Therefore, therapeutic strategies that target the distinct mitochondrial metabolic features of CSCs and DTPs, such as disrupting the AMPK–OXPHOS axis, inhibiting mitophagy, or decoupling metabolic signaling from DNA repair, represent a promising approach to overcoming chemoresistance in CRC.

### Signaling—metabolic crosstalk in CRC: a dual-track mechanism of resistance to targeted therapy

Precision therapies targeting EGFR, BRAF, and MEK have offered new hope for patients with CRC [74]. However, clinical efficacy is often limited by a pattern of short-term response followed by long-term resistance [74]. Emerging evidence indicates that reactivation of oncogenic signaling pathways is frequently accompanied by parallel reprogramming of metabolic networks, forming a coordinated “dual-track” mechanism of resistance driven by signaling-metabolism crosstalk [75].

In BRAF V600E-mutant CRC, tumor cells often exhibit an initial response to BRAF inhibitors, but frequently develop resistance through reactivation of the MAPK pathway, commonly via KRAS amplification or MEK mutations [76]. During this adaptive process, cellular metabolic phenotypes shift accordingly by either increasing OXPHOS flux or enhancing glycolytic dependence to meet the energy demands imposed by sustained oncogenic signaling [75].

Within this context, the MPC serves as a critical metabolic gatekeeper. Loss of MPC function leads to cytosolic pyruvate accumulation and impairs entry into the tricarboxylic acid (TCA) cycle, thereby shifting cells toward a glycolytic phenotype and promoting resistance to OXPHOS inhibition [18]. Concurrently, activation of the Nrf2–GSH axis enhances the cellular antioxidant capacity, enabling tumor cells to maintain redox homeostasis under targeted therapy–induced oxidative stress, thus facilitating metabolic escape [77].

Recent studies have shown that the RNA-binding protein RALY promotes OXPHOS activation and mitochondrial metabolic remodeling by regulating microRNA processing, thereby enhancing the adaptive capacity of CRC cells to EGFR or MEK inhibition [9]. Complementing this, PINK1-dependent mitophagy is upregulated during treatment to eliminate damaged mitochondria, preserve mitochondrial network integrity, and sustain both metabolic output and signal transduction fidelity [78].

Together, resistance to targeted therapy in CRC reflects a co-evolution of signaling reactivation and mitochondrial metabolic reprogramming [79]. Combined interventions targeting MPC dysfunction, ROS buffering systems such as the Nrf2–GSH axis, or mitophagy pathways including PINK1, may disrupt the self-sustaining signal–metabolism circuit and offer a strategy to delay or reverse acquired therapeutic resistance.

### Decoding immune resistance in MSS CRC: the role of metabolic–immune crosstalk

Immune checkpoint inhibitors (ICIs) have demonstrated significant efficacy in patients with microsatellite instability-high (MSI-H) CRC, yet their therapeutic benefit remains limited in the majority of cases, which are microsatellite stable (MSS) [80]. One of the central barriers to effective immune response in MSS CRC is the metabolically driven immunosuppressive tumor microenvironment [81].

MSS CRC cells often exhibit elevated glycolytic and OXPHOS activity, leading to excessive consumption of glucose and oxygen within the tumor microenvironment (TME), thereby depriving effector immune cells such as CD8⁺ T cells of essential metabolic resources [82]. Concurrently, lactate accumulation contributes to an acidic tumor microenvironment that impairs T cell effector function, induces exhaustion, and suppresses proliferation [83]. Under hypoxic conditions, HIF-1α promotes PD-L1 upregulation, reinforcing immune evasion through both metabolic and immunoregulatory mechanisms [84].

In addition, aberrant activation of the WNT/β-catenin signaling pathway represents another immune resistance mechanism in MSS CRC. This pathway not only suppresses CD8⁺T cell infiltration but also downregulates major histocompatibility complex class I (MHC I) expression and inhibits interferon-gamma (IFN-γ) signaling, thereby undermining antitumor immune responses at multiple levels [85].

Recent studies have revealed that the tumor microbiome also plays a critical role in mediating coordinated metabolic and immune resistance. A study published in Nature Cancer reported that *Fusobacterium nucleatum* impairs antitumor immunity by modulating NAD⁺ metabolism and upregulating PD-L1 expression [86]. Moreover, the tumor microbiota is positively correlated with mitochondrial metabolic activity [87, 88], forming a “microbiota–metabolism–immunity axis” that underpins a multidimensional resistance network and offers new insights for next-generation tumor microenvironment–targeted strategies.

Building on these findings, studies have shown that the active mitochondrial network in CRC cells exerts a physical “metabolic occupancy effect” that impedes immunological synapse formation, while the associated release of ROS further suppresses immune cell function. For example, the team led by Hai Wang has developed nanoscale mitochondrial inducers (mitoNIDs) that selectively eliminate mitochondria in tumor cells, reduce ROS levels in the TME, and restore the metabolic activity and function of CD8⁺T cells [89].

Based on these mechanisms, several emerging strategies aim to overcome metabolic–immune cooperative resistance and reprogram the tumor microenvironment to enhance immunotherapy efficacy. These approaches include the combined use of STING agonists, Nrf2 inhibitors, lactate metabolism blockers, and mitoNIDs, which collectively alleviate immunosuppressive metabolic states, restore T cell function [89–91], and improve the responsiveness of MSS CRC to immune ICIs.

### Metabolic reprogramming and CRC metastasis: mechanistic insights and therapeutic implications

Beyond its established role in therapy resistance, metabolic reprogramming is increasingly recognized as a critical driver of CRC metastasis, a major cause of poor patient prognosis [92, 93]. Growing evidence suggests that CRC cells exploit specific metabolic adaptation mechanisms to cope with the microenvironments of distant organs, thereby enhancing their invasiveness and metastatic colonization capacity.

Montero-Calle et al. found that highly metastatic CRC cells primarily rely on glutamine-driven TCA cycle activity to sustain energy production, whereas low-metastatic cells tend to depend more on glycolysis [94]. These findings suggest that metabolic heterogeneity may serve as a key biomarker for predicting metastatic potential. Building on this, Wang et al. further demonstrated that MTA1 functions as a novel regulator of ATP synthase, promoting liver metastasis by enhancing OXPHOS activity and shifting glucose metabolic preferences [95]. Moreover, dysregulated lipid metabolism networks such as ACSL/SCD also drive EMT and migratory properties, and their pharmacologic inhibition selectively impairs CRC cell viability, further broadening the landscape of metabolic vulnerabilities in metastatic CRC [96].

Taken together, these findings highlight that metabolic reprogramming not only provides energy and biosynthetic precursors for metastasis but also enables cancer cells to acquire invasive and migratory phenotypes. Targeting key metabolic nodes, especially in combination with immune checkpoint inhibitors such as anti-PD-1, holds great promise for achieving novel therapeutic breakthroughs in the management of metastatic CRC.

## Central regulators of mitochondrial reprogramming: coordinated control by metabolic enzymes, spatiotemporal heterogeneity, and post-transcriptional networks

**Fig. 3:**
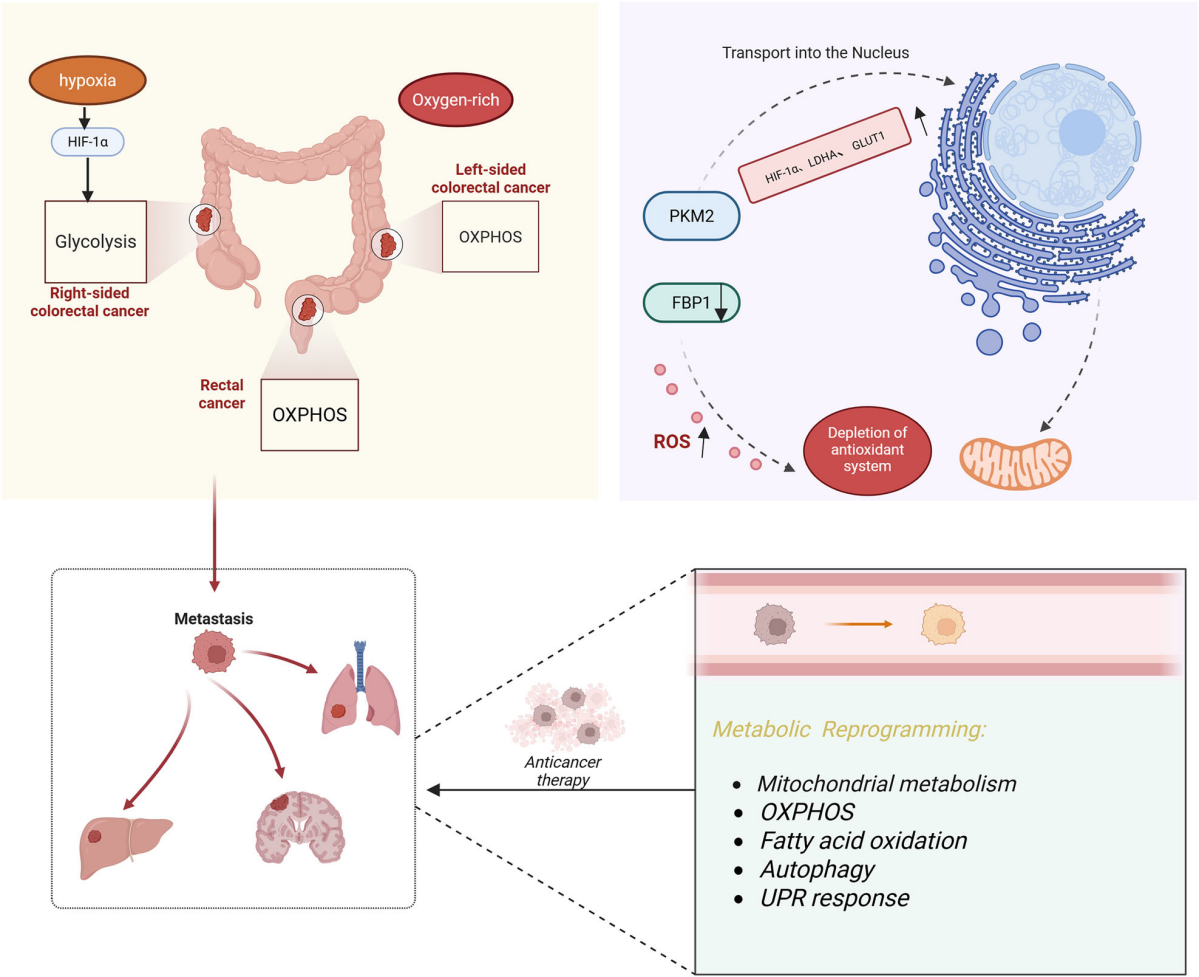
Metabolic heterogeneity and mitochondrial reprogramming in CRC. Colorectal tumors exhibit spatial metabolic diversity, with right-sided cancers favoring glycolysis and left-sided/rectal cancers relying on OXPHOS. Key enzymes like PKM2 and FBP1 regulate redox balance and gene expression, promoting ROS accumulation and resistance. Therapy-induced stress drives mitochondrial adaptations, supporting metastasis and drug resistance through enhanced oxidative metabolism.

Building on previous sections that examined how CRC develops resistance through mitochondrial metabolic reprogramming, this section explores three key regulatory mechanisms that sustain such adaptability. We highlight the non-canonical roles of multifunctional metabolic enzymes in signaling and redox control, the impact of spatial and temporal metabolic heterogeneity in shaping mitochondrial function, and the post-transcriptional regulation of mitochondrial metabolism by miRNAs in CRC. Together, these insights help elucidate the origins of metabolic reprogramming and inform the development of targeted therapeutic strategies (Fig. 3).

### Multifunctional metabolic enzymes in mitochondrial regulation: initiating signals of drug resistance in CRC

Within the complex metabolic adaptation network of CRC, certain conventional metabolic enzymes are increasingly recognized as critical regulatory nodes that link energy flux to therapeutic response, owing to their moonlighting properties, which involve multiple non-canonical functions beyond their enzymatic roles [94]. These enzymes not only maintain basal metabolic flux through their catalytic activity but also participate in epigenetic regulation, transcription factor activation, redox homeostasis, and the modulation of cell death pathways, thereby acting as upstream drivers of mitochondrial metabolic reprogramming [95].

One of the most well-characterized examples is pyruvate kinase isoform M2 (PKM2). While PKM2 predominantly exists as a catalytically active tetramer in normal tissues, it tends to adopt a low-activity dimeric form in CRC and various other tumor types. This conformational shift impairs the conversion of phosphoenolpyruvate (PEP), leading to the accumulation of glycolytic intermediates that fuel anabolic biosynthesis [96]. More importantly, PKM2 can translocate into the nucleus, where it regulates the transcription of metabolic genes such as HIF-1α, LDHA, and GLUT1, thereby enhancing glycolysis, suppressing OXPHOS, and establishing a metabolic “firewall” against therapeutic stress [97].

Another critical enzyme is fructose-1,6-bisphosphatase 1 (FBP1), a rate-limiting enzyme in gluconeogenesis that is markedly downregulated in CRC [98]. Loss of FBP1 not only sustains elevated glycolytic flux, but also destabilizes the mitochondrial membrane potential, increases ROS levels, and impairs activation of antioxidant defenses such as SOD2 [99]. Beyond its metabolic role, FBP1 exhibits non-canonical functions in regulating mitosis, controlling cell cycle progression, and modulating c-Myc-dependent transcriptional programmes, thereby contributing to the persistence of drug-resistant cell populations on multiple fronts [100].

Notably, the research paradigm of multifunctional enzymes has increasingly extended into the field of biomimetic materials. Dong et al. developed a copper-based metal-organic framework (MOF) nanozyme with dual peroxidase- and oxidase-like activities that exerts potent metabolic intervention in CRC [101]. By inducing ROS accumulation, modulating mitochondrial membrane potential, and activating oxidative stress-related pathways, the nanozyme suppresses tumor proliferation and restores sensitivity to chemotherapeutic agents such as 5-FU [101]. This study highlights the potential of enzyme-mimicking nanomaterials in replicating multifunctional enzyme activity and supports material-based strategies for targeting cancer metabolism.

Collectively, multifunctional metabolic enzymes act as pivotal hubs linking metabolic reprogramming to therapeutic resistance, serving as upstream initiators of signaling cascades that drive drug tolerance in CRC. Whether through modulating central glycolytic pathways, disrupting redox homeostasis, or reshaping transcriptional programs, their functions extend well beyond the classical roles of conventional enzymes. In the future, selectively targeting the non-catalytic functions of these enzymes may represent a novel strategy for precision metabolic intervention.

### How metabolic heterogeneity shapes mitochondrial function and therapeutic resistance in CRC

CRC exhibits a metabolically heterogeneous landscape, shaped by tumor location, evolutionary stage, and the surrounding microenvironment [102]. It constitutes a fundamental biological basis underlying variability in therapeutic response and the emergence of drug resistance. For instance, Tao et al. identified mitophagy as an intrinsic vulnerability in CRC cells subjected to statin treatment, revealing how disruption of mitochondrial homeostasis, shaped by distinct metabolic contexts, leads to cell death and therapeutic sensitivity [103]

Spatial heterogeneity across colorectal tumor sites underlies distinct metabolic phenotypes [104]. Right-sided colon cancers are frequently characterized by high microsatellite instability (MSI-H) and pronounced immune infiltration, accompanied by a glycolysis-dominant metabolic profile. In contrast, left-sided colon and rectal tumors exhibit elevated mitochondrial activity and a greater reliance on OXPHOS [105]. Intra-tumoral oxygen gradients exert a decisive influence on metabolic pathway selection. Hypoxic regions activate HIF-1α, driving a shift toward glycolytic metabolism, whereas oxygen-rich zones sustain robust mitochondrial respiration and energy production [106]. This spatial metabolic heterogeneity confers marked differences in therapeutic responsiveness among cancer cells residing in distinct tumor niches.

Temporally, CRC exhibits distinct metabolic evolution during progression from primary to metastatic disease [107]. Studies have shown that glucose uptake decreases in advanced CRC, whereas levels of oxidative metabolites such as glutamate and pyruvate increase, indicating a metabolic shift from glycolysis toward a mitochondria-centered hybrid phenotype [6]. Under therapeutic stress, subpopulations enhance mitochondrial respiration, FAO, and TCA cycle activity, promoting redox balance and bioenergetic resilience that underpin chemoresistance and metastatic potential [108].

In organ-specific metastatic lesions, metabolic heterogeneity is also pronounced. Liver metastases, for instance, often display elevated lipid metabolic activity and enhanced antioxidant capacity, characterized by high mitochondrial density and intact electron transport chain (ETC) function—features that help counteract chemotherapy-induced ROS accumulation [109]. A study further revealed that the exonuclease MYG1 promotes tumor adaptation and progression in CRC by coordinating nuclear–mitochondrial signaling, thereby boosting both glycolysis and mitochondrial function [68]. These findings underscore mitochondrial metabolic plasticity as a key driver of tumor heterogeneity and therapy resistance.

Metabolic heterogeneity in CRC not only compounds therapeutic challenges but also profoundly influences cell survival and drug responsiveness through the reprogramming of mitochondrial function. Advances in metabolomics, single-cell multi-omics, and mitochondrial functional imaging offer unprecedented resolution to delineate the spatiotemporal dynamics of tumor metabolism. Such insights are poised to inform the development of region-specific and adaptively tuned metabolic targeting strategies.

### miRNAs as post-transcriptional regulators of mitochondrial reprogramming in CRC

Beyond metabolic enzyme regulation and tumor microenvironmental heterogeneity, miRNAs function as pivotal post-transcriptional regulators that orchestrate mitochondrial metabolic reprogramming in CRC. Through multifaceted mechanisms, miRNAs integrate oncogenic signaling with extrinsic stress responses, positioning them as key modulators of metabolic adaptation.miRNAs exert fine-tuned control over a wide array of metabolic pathways, including glycolysis, OXPHOS, fatty acid and amino acid metabolism, mitochondrial dynamics, and redox homeostasis [9]. By reshaping the metabolic phenotype of tumor cells, miRNAs enhance their survival, metastatic potential, and resistance to therapy [110].

In CRC, miR-21 is one of the most prominent oncogenic miRNAs [111]. It promotes chemoresistance by suppressing PTEN and activating the PI3K/AKT/mTOR signaling cascade, which in turn impairs mitochondrial respiration while enhancing glycolysis and lactate accumulation. These metabolic shifts support the survival of tumor cells under chemotherapeutic stress and contribute to treatment resistance [111, 112]. Similarly, hypoxia-inducible factor 1-alpha (HIF-1α) upregulates the miR-23a/24 cluster, driving a metabolic shift toward glycolysis under hypoxic conditions and thereby accelerating cancer progression [112]. miR-23a and miR-23b have also been shown to downregulate glutaminase (GLS), thereby disrupting glutamine metabolism. However, their precise roles in the regulation of drug resistance remain to be fully elucidated [113]. More recently, miR-141-3p, downregulated by lncRNA HIF1A-AS2, has been shown to promote CRC metabolic reprogramming and proliferation through FOXC1 activation, highlighting additional post-transcriptional networks under hypoxic stress [114]. Beyond intracellular regulation, extracellular miRNAs also contribute to metabolic adaptation. miR-1246, released via exosomes from CRC cells, can reprogram tumor-associated macrophages (TAMs) toward a pro-tumor phenotype by altering their metabolic state, thereby supporting CRC growth and immune evasion [115].

Meanwhile, tumor-suppressive miRNAs play a critical role in constraining the metabolic plasticity of cancer cells. miR-137, for instance, targets the glutamine transporter SLC1A5, thereby reducing glutamine uptake and impairing TCA cycle replenishment [116, 117]. Its epigenetic silencing in CRC leads to enhanced glutaminolysis and therapeutic resistance. miR-181d, on the other hand, reprograms circadian and metabolic regulators such as CRY2 and FBXL3, establishing a feedback loop with c-Myc to promote CRC progression [118]. miRNAs also control mitochondrial dynamics and redox balance, both essential for metastatic potential and treatment adaptation [119]. miR-155 has been shown to suppress Parkin-mediated mitophagy, resulting in the accumulation of dysfunctional mitochondria and apoptotic resistance [120, 121]. In contrast, miR-27a promotes mitophagy by targeting USP14, destabilizing BAG4, and enhancing 5-FU sensitivity in MSI-high CRC [122]. Additionally, mitochondria-localized miRNAs (mito-miRs), such as miR-124, regulate mitochondrial gene expression and transcriptional machinery, thereby contributing directly to chemoresistance and metabolic adaptation [110].

Together, miRNAs drive mitochondrial metabolic reprogramming in CRC by modulating energy production, redox homeostasis, and tumor microenvironment responses to stress. This underscores their central role in metabolic plasticity and highlights the promise of miRNA-targeted therapies combined with metabolic inhibitors in precision oncology.

## Mitochondrial metabolic heterogeneity in CRC: implications for therapeutic divergence and precision intervention

Building on earlier discussions of how mitochondrial metabolism contributes to resistance in CRC, driven by metabolic enzymes, intratumoural heterogeneity, and post-transcriptional regulatory networks, this section highlights the clinical relevance of metabolic divergence. We further examine how such heterogeneity influences treatment response and outline precision-targeting strategies aimed at overcoming adaptive metabolic resistance.

### How metabolic heterogeneity shapes mitochondrial adaptation and differential therapeutic response

**Fig. 4:**
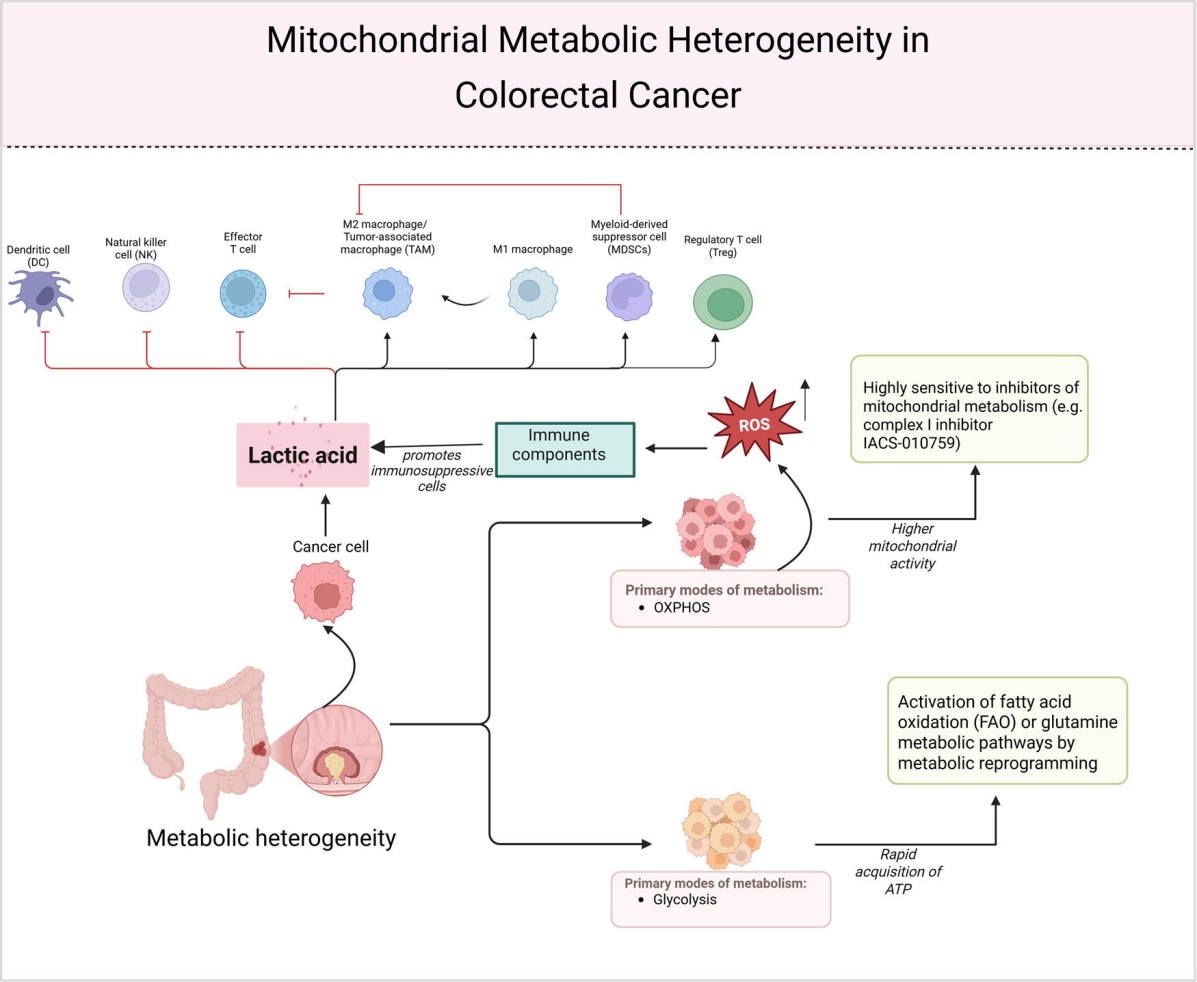
Mitochondrial metabolic heterogeneity shapes immune regulation and therapeutic response in CRC. Metabolic heterogeneity in CRC drives functional divergence among tumor subpopulations, with OXPHOS-dominant cells showing high mitochondrial activity and glycolysis-dominant cells rapidly generating ATP. These distinct states influence therapeutic sensitivity and immune evasion. OXPHOS-high cells produce ROS and are sensitive to mitochondrial inhibitors (e.g., IACS-010759), while glycolytic cells adapt via fatty acid or glutamine metabolism. Lactic acid accumulation modulates immune cell polarization, promoting an immunosuppressive tumor microenvironment and reinforcing resistance.

Metabolic heterogeneity within CRC reflects more than spatial differences in energy pathways; it also embodies the distinct adaptive capacities and metabolic plasticity of diverse cellular subpopulations in response to stress [123] (Fig. 4).

Recent studies have revealed that OXPHOS-dominant subpopulations, while reliant on mitochondrial energy production, exhibit marked sensitivity to complex I inhibitors such as IACS-010759. Inhibition of the respiratory chain rapidly induces mitochondrial membrane potential collapse and apoptosis in these cells [124]. In contrast, glycolysis-driven subpopulations can evade metabolic stress by reprogramming their metabolism through FAO and glutamine utilization, giving rise to highly treatment-resistant cellular subsets [125].

Moreover, metabolic heterogeneity extends its influence through paracrine effects mediated by metabolic by-products, profoundly shaping the tumor immune microenvironment [125]. Lactate secreted by CRC cells promotes the polarization of TAMs toward an M2-like immunosuppressive phenotype, impairs CD8⁺ T cell function, and reduces the efficacy of ICIs [126]. In parallel, regions enriched in OXPHOS activity often exhibit enhanced ROS regulation, further reinforcing the immunosuppressive barrier.

Notably, a study published in nature metabolism reported that regions with high OXPHOS activity in metastatic CRC are often enriched with immunosuppressive cells and TAMs, suggesting a coupling between mitochondrial metabolic states and immune symbiosis [6]. This metabolic–immune interplay not only reinforces therapeutic resistance but may also contribute to the establishment of a “metabolic protective niche,” providing a sanctuary within the multicellular tumor ecosystem that shields resistant subpopulations from treatment-induced stress.

In summary, metabolic heterogeneity in CRC is not merely a passive adaptive feature but actively shapes differential treatment responses. A deeper understanding of how distinct metabolic subtypes function under stress and influence the tumor microenvironment will inform the development of more selective and subtype-specific metabolic interventions. This is particularly relevant for combination strategies involving immunotherapy and metabolic pathway targeting, offering new avenues to overcome therapeutic resistance.

### Overcoming metabolic heterogeneity-driven resistance: multidimensional interventions and translational advances

To overcome resistance driven by metabolic heterogeneity in CRC, current research is advancing multi-layered and pathway-diverse intervention strategies [125]. The central objectives are to disrupt cancer cell metabolic plasticity, prevent interconversion between distinct metabolic states, and concurrently reprogram the immunometabolic landscape to restore therapeutic sensitivity [127].

One strategic approach involves combining metabolic inhibitors with key signaling pathway modulators to establish a dual-targeted metabolic–signaling axis. For instance, co-administration of the OXPHOS inhibitor IACS-010759 with PI3K/Akt/mTOR inhibitors concurrently disrupts energy production and survival signaling, substantially reducing the tumor’s capacity for metabolic adaptation [128].

Another strategy focuses on identifying and targeting specific metabolic dependencies, a concept referred to as “metabolic vulnerability therapy.” In cell subpopulations with elevated OXPHOS activity and glutamine-driven TCA cycle flux, the combined use of glutamine metabolism inhibitors such as CB-839 with oxidative stress inducers, including doxorubicin or isothiocyanates, markedly increases intracellular ROS levels. This surge in oxidative stress disrupts mitochondrial function and triggers programmed cell death, enabling the selective elimination of metabolically adaptable tumor cells [43, 129].

Advances in high-resolution technologies such as single-cell omics, spatial metabolomics, and imaging mass spectrometry have progressively unveiled the metabolic heterogeneity landscape within CRC [130, 131]. Recent studies have shown that certain metastatic subpopulations exhibit a strong dependence on glutamine-driven TCA cycle activity, whereas others highly express FAO enzymes, maintain low levels of ROS, and display enhanced migratory capacity and immune evasion [40]. Artificial intelligence (AI)-assisted spatial metabolic imaging and response modeling have been employed to dynamically predict drug responsiveness across distinct tumor regions, providing a robust foundation for the development of metabolism-guided combination strategies [132].

As mitochondrial-targeted strategies advance toward clinical application, functional biomarkers of mitochondrial activity are emerging as important tools for predicting treatment response. For example, mtDNA copy number correlates with OXPHOS activity and may indicate potential sensitivity to mitochondrial inhibitors [133]; mitochondrial membrane potential (e.g., via JC-1 staining) reflects shifts in cellular energy state; the OCR/ECAR ratio serves as an indicator of metabolic preference and can help guide rational combination strategies; and ROS levels may predict responsiveness to inhibitors of antioxidant pathways [134]. These biomarkers are dynamically measurable and functionally relevant, providing a practical framework for personalized metabolic intervention.

Taken together, metabolic heterogeneity is both a driver of therapeutic resistance and a promising entry point for precision intervention in cancer. Integrating mitochondrial functional profiling, multi-omics spatial mapping, and multi-targeted therapies could enable a paradigm shift toward individualized treatments guided by metabolic vulnerabilities.

## Clinical translation: from mitochondrial metabolic mechanisms to precision therapeutic pathways

Advances in understanding mitochondrial metabolic reprogramming in CRC are accelerating its translation into clinical practice. Emerging developments in metabolic diagnostics, targeted therapies, novel agents, and combination strategies are positioning metabolism-driven precision medicine as a new frontier in CRC care [24]. This section outlines key translational pathways across diagnosis, intervention, drug development, and clinical trial design.

### Diagnostic innovation from a metabolic perspective

In the realm of precision diagnostics, metabolism-associated technologies are increasingly enhancing the early detection and molecular stratification of CRC [135]. Among these, circulating tumor DNA (ctDNA) has emerged as a minimally invasive biomarker for monitoring therapeutic response and detecting minimal residual disease [136]. Recent studies also suggest its potential to predict metabolically driven resistance subtypes. Notably, integrating ctDNA analysis with the detection of mutations associated with metabolic dysregulation, such as KRAS and BRAF, has been shown to enhance diagnostic sensitivity [137].

At the same time, metabolomics is emerging as a complementary tool in molecular diagnostics. By profiling small-molecule metabolites in biofluids such as plasma and urine, researchers have identified distinct metabolic fingerprints associated with CRC, including glutamate, pyruvate, and fatty acid derivatives, which correlate with specific tumor metabolic subtypes [138, 139]. A study further reported that integrating metabolomic data with gut microbiome features significantly improves the sensitivity of early CRC detection—particularly in high-risk populations [140].

At the imaging level, metabolic imaging modalities such as positron emission tomography (PET), functional magnetic resonance imaging (fMRI), and emerging techniques like magnetic resonance spectroscopic imaging (MRSI) are being employed for non-invasive assessment of tumor metabolic activity and mitochondrial function [141, 142]. These approaches provide quantitative metrics to support tumor staging, prognostic evaluation, and monitoring of treatment response.

### Therapeutic targeting of mitochondrial metabolic dependencies

CRC cells sustain a high metabolic state and therapeutic resistance by activating multiple mitochondrial pathways, including the TCA cycle, OXPHOS, FAO, and one-carbon metabolism [18]. Targeting these mitochondrial dependencies has emerged as a promising therapeutic strategy in CRc [6].

Inhibition of the ETC represents one of the most extensively studied strategies to date. Complex I inhibitors, such as IACS-010759 and BAY87-2243, have demonstrated potent efficacy in preclinical models of CRC subtypes with high OXPHOS dependence [143].

In the context of lipid metabolism, targeting pathways such as ether lipid synthesis, fatty acid synthase (FASN), or the ACLY/ACC axis has been shown to induce mitochondrial stress and ROS accumulation, thereby enhancing apoptotic responses [144]. Glutamine metabolism inhibition, using agents such as CB-839, has shown potential to suppress TCA cycle-mediated energy production and disrupt redox homeostasis, particularly in CRC subtypes resistant to 5-FU and oxaliplatin [145].

Furthermore, studies have shown that mitochondrial-targeted nanodrugs enhance the sensitivity of cancer cells to chemotherapy drugs by intervening in mitochondrial function, further emphasizing the emerging therapeutic strategy of reversing drug resistance through mitochondrial metabolic reprogramming [146]. For example, a tri-functional copper-based MOF nanozyme developed by Dong et al.not only generates ROS but also modulates the redox environment, overcoming chemotherapy resistance in CRC and providing a new therapeutic strategy for future clinical applications [101].

Wang et al.further proposed a mitochondria-targeted biomimetic nanomedicine (OXA@Exo-RD), which employs an exosome-mediated sequential delivery strategy to achieve precise mitochondrial delivery of oxaliplatin in drug-resistant CRC cells. This nanomedicine induces mitochondrial membrane potential collapse, ATP depletion, and excessive accumulation of ROS, ultimately triggering mitochondria-dependent apoptosis, thereby effectively reversing oxaliplatin resistance [146]. This study not only validates the feasibility of the “mitochondrial dysfunction-induced cell death” strategy, but also demonstrates the translational potential of biomimetic drug delivery systems in metabolism-targeted therapy.

### Development of novel therapeutics targeting mitochondrial metabolism

In recent years, an increasing number of mitochondria-targeted therapeutic candidates have entered preclinical development or clinical evaluation, showing promising anti-CRC potential [146] (Table 1).

**Table 1:** Novel mitochondria-targeting drugs in CRC.

Drug/compound	Target/mechanism	Effects	Development stage	References
MitoDFO	Targets mitochondrial iron metabolism; inhibits [Fe–S] cluster/heme biosynthesis	Induces mitophagy, suppresses tumor growth/metastasis; selectively impairs respiratory chain complexes	Preclinical (in vitro and in vivo studies)	[189]
Glycyrrhetinic acid (GA)	Inhibits SHMT2, blocking mitochondrial one-carbon metabolism	Reduces nucleotide synthesis, inhibits CRC proliferation; enhances efficacy via epigenetic regulation	Phase I (CRC)	[190]
Pyrrolo[3,2-d]pyrimidine derivatives	Dual inhibition of SHMT2 and MTHFD2	Disrupts folate cycle, induces apoptosis; synergizes with 5-FU	Preclinical	[147, 191]
IACS-010759	Complex I inhibitor (OXPHOS suppression)	Causes S-phase arrest, depletes aspartate; synergizes with GOT1 inhibitors	Phase II (combination)	[148]
Idasanutlin (RG-7388)	Activates p53, inhibits HSF1-HSP90 axis	Combines with HSP90 inhibitors to destabilize AKT/cRAF, promoting cell death	Phase I/II (combination)	[149]
Palbociclib	CDK4/6 inhibitor; blocks p21-CDK4/6-MAPK-HSF1 pathway	Enhances metabolic targeting in p53-deficient CRC when combined with HSP90 inhibitors; promotes cell cycle arrest and cell death	Phase III (combination)	[150]
Ganetespib/Onalespib	HSP90 inhibitor targeting APC and related client proteins (e.g., mutant p53, RTKs)	Disrupts client proteins, overcomes resistance with p53 activators, and targets the Wnt signaling pathway in CRC	Phase II (combination)	[149]
TRAP1 inhibitors (e.g., DN-214)	Inhibits mitochondrial HSP TRAP1, impairing respiratory chain assembly, and disrupting mitochondrial function	Induces mitochondrial stress and synergizes with chemotherapy by enhancing metabolic stress in cancer cells	Preclinical	[151]
Ribitol	Modulates glycolysis/nucleotide metabolism (target unknown) in breast cancer cells	Promotes apoptosis in breast cancer cells in combination with Shikonin	Exploratory	[152]
GOT1 inhibitors	Targets GOT1, modulating glucose metabolism by increasing glucose dependence	Alters cell metabolism, increases glucose dependence in cancer cells	Preclinical	[153]
IMT1	Mitochondrial RNA polymerase (POLRMT) inhibitor	Inhibits mitochondrial transcription, suppresses CRC cell growth	Preclinical	[154]
Divarasib	Small molecule targeting KRAS G12C mutations, tested in combination with cetuximab	Inhibits CRC cell proliferation and enhances response to treatment	Phase 1b clinical trials	[155]

Table 1 summarizes some novel mitochondria-targeting compounds in the treatment of CRC. It outlines the mechanism of action, effects, development stage, and relevant references for each drug.

These agents disrupt key mitochondrial functions through diverse mechanisms. For instance, mitoDFO impairs iron–sulfur cluster and heme biosynthesis [147], while glycyrrhetinic acid (GA) and pyrrolo[3,2-d]pyrimidine derivatives inhibit one-carbon metabolism [148–150]. The OXPHOS inhibitor IACS-010759 has also shown potent anti-tumor activity in CRC models [151].

Several agents, including Idasanutlin and Palbociclib, have demonstrated enhanced therapeutic efficacy when combined with HSP90 inhibitors, highlighting the potential of targeting mitochondrial stress response pathways [152, 153]. Additional therapeutic strategies focus on the modulation of metabolic cofactors such as Ribitol and GOT1 inhibitors [154, 155], as well as the inhibition of mitochondrial chaperones, including TRAP1 [156]. Collectively, these approaches underscore the growing recognition of mitochondrial vulnerabilities as viable targets in CRC therapy.

Recent efforts have expanded beyond traditional mitochondrial targets to include upstream oncogenic drivers that influence mitochondrial function.IMT1 exemplifies the potential of disrupting mitochondrial gene expression, while Divarasib reflects efforts to integrate oncogene-targeted therapies into mitochondria-centered treatment strategies. Together, these agents represent a broader shift toward combining mitochondrial vulnerabilities with upstream molecular drivers to improve CRC outcomes [157, 158].

These novel mitochondrial-targeted agents show substantial promise in the treatment of CRC. By interfering with key mitochondrial metabolic pathways, these therapeutics effectively suppress tumor growth and metastasis, offering new avenues for CRC management and precision interventions.

### Combination therapies: multimodal strategies to overcome drug resistance

CRC is characterized by high metabolic plasticity, which often undermines the durability of single-target therapies. Under treatment pressure, tumor cells rapidly activate alternative pathways, driving resistance. Consequently, combination strategies that concurrently target metabolic adaptation and bypass signaling are emerging as effective approaches to enhance efficacy and delay resistance. Multiple clinical studies have shown that integrating metabolic modulation with immunotherapy, chemotherapy, or targeted agents can significantly improve outcomes (Table 2).

**Table 2:** CRC multi-pathway combination therapies for overcoming resistance.

Combination strategy	Drug combination example	Target/mechanism	Effects	Development stage	References
Metabolic inhibition + Signal pathway inhibition	Trametinib (MEK inhibitor) + 5-FU (metabolic inhibition)	Inhibit the MAPK pathway, sensitize tumors to 5-FU	Clinical studies have shown this combination improves pathological response to neoadjuvant chemoradiation	Phase I clinical trial	[156]
Anti-angiogenesis + Immunotherapy	Cabozantinib (anti-c-Met) + Cetuximab (anti-EGFR)	Inhibit tumor angiogenesis and modulate the immune microenvironment	Enhances immune response and shows efficacy in chemo-refractory MSS CRC	Phase II clinical trial	[157]
Immunotherapy + Targeted therapy	Nivolumab (PD-1 inhibitor) + Regorafenib (multi-kinase inhibitor)	Enhance immune infiltration and activity in MSS CRC	Shows partial clinical activity in MSS CRC, especially in patients without liver metastasis	Phase II clinical trial	[158]
Ferroptosis induction + Chemotherapy sensitization	SLC7A11 inhibitor (e.g., Erastin) + FOLFOX (chemotherapy regimen)	Inhibit SLC7A11 to reduce GSH synthesis, induce lipid peroxidation, and ferroptosis	Enhances chemosensitivity, reverses oxaliplatin resistance in CRC (preclinical)	Preclinical studies	[159]
Apoptosis induction + Cell cycle inhibition	DR5-targeting ADC (Oba01) + CDK inhibitor (e.g., Abemaciclib)	Oba01 induces apoptosis via MMAE, while CDK inhibitors arrest the cell cycle; the combination synergistically enhances CRC suppression.	Synergistically suppresses CRC tumor growth; overcomes resistance in advanced and chemo-refractory MSS CRC	Preclinical studies	[160]
Metabolic disruption + T cell immunotherapy	mitoNIDs (mitochondria-targeted nanoinducers) + T cell-based immunotherapy	mitoNIDs selectively induce mitochondrial degradation in tumor cells, enhancing immunogenicity and facilitating T cell-mediated cytotoxicity	Boosts antitumor immunity, enhances T cell infiltration and efficacy in otherwise resistant tumors	Preclinical studies	[89]

This table summarizes selected therapeutic combinations involving metabolic inhibition with signal transduction blockade or immunotherapy. These approaches are currently under clinical evaluation to overcome resistance in CRC.

For example, the MEK inhibitor trametinib combined with 5-FU has been shown to synergistically suppress the MAPK pathway and enhance the response to neoadjuvant chemoradiotherapy [156]. The combination of cabozantinib (anti-c-MET) with durvalumab (anti-PD-L1) can inhibit tumor angiogenesis and modulate the immune microenvironment, demonstrating promising efficacy in MSS CRC patients refractory to chemotherapy [157]. Co-administration of nivolumab and regorafenib has been reported to enhance immune infiltration, with partial responses particularly evident in patients without liver metastases [158]. In addition, SLC7A11 inhibitors such as erastin, which induce ferroptosis, significantly improve oxaliplatin sensitivity when used in combination with FOLFOX, highlighting the potential of metabolic interventions to reverse chemoresistance [159].

The introduction of nanotechnology has further advanced dual strategies that couple physical metabolic disruption with immune activation. Recent studies have shown that mitochondria-targeted nanoinducers (mitoNIDs) can selectively impair mitochondrial function in cancer cells, disrupting energy metabolism and redox balance [89]. This intervention markedly enhances the antitumor efficacy of CAR-T cells, CD8⁺ T cells, and mRNA vaccines, offering a promising breakthrough for overcoming resistance in immunotherapy-refractory solid tumors such as MSS CRC.

Building on this rationale, recent preclinical findings underscore the promise of combining targeted apoptosis inducers with cell-cycle inhibitors. For instance, Zhou et al. showed that a DR5-targeting antibody-drug conjugate combined with a CDK inhibitor synergistically suppressed tumor growth in advanced CRC models, including chemotherapy-resistant settings. This approach not only potentiated apoptosis but also blocked compensatory survival pathways, supporting its potential to overcome resistance in MSS CRC [160].

Such multimodal combinations not only leverage mechanistic complementarity to enhance efficacy, but also reduce toxicity and delay relapse, yielding a more durable and controllable therapeutic response. By simultaneously targeting multiple pathways, impairing mitochondrial function, and engaging immune mechanisms, this strategy is defining the core framework of next-generation precision therapies for CRC.

As research progresses from molecular subtyping and metabolic mechanisms to therapeutic combinations and clinical validation, mitochondrial metabolic reprogramming has evolved from a fundamental biological question into a cornerstone of precision intervention. With the continued integration of interdisciplinary technologies and the acceleration of clinical translation, metabolic targeting in CRC is entering a new era of broad clinical applicability.

## Future directions and clinical translation challenges—towards mitochondria-guided precision treatment for CRC

**Fig. 5:**
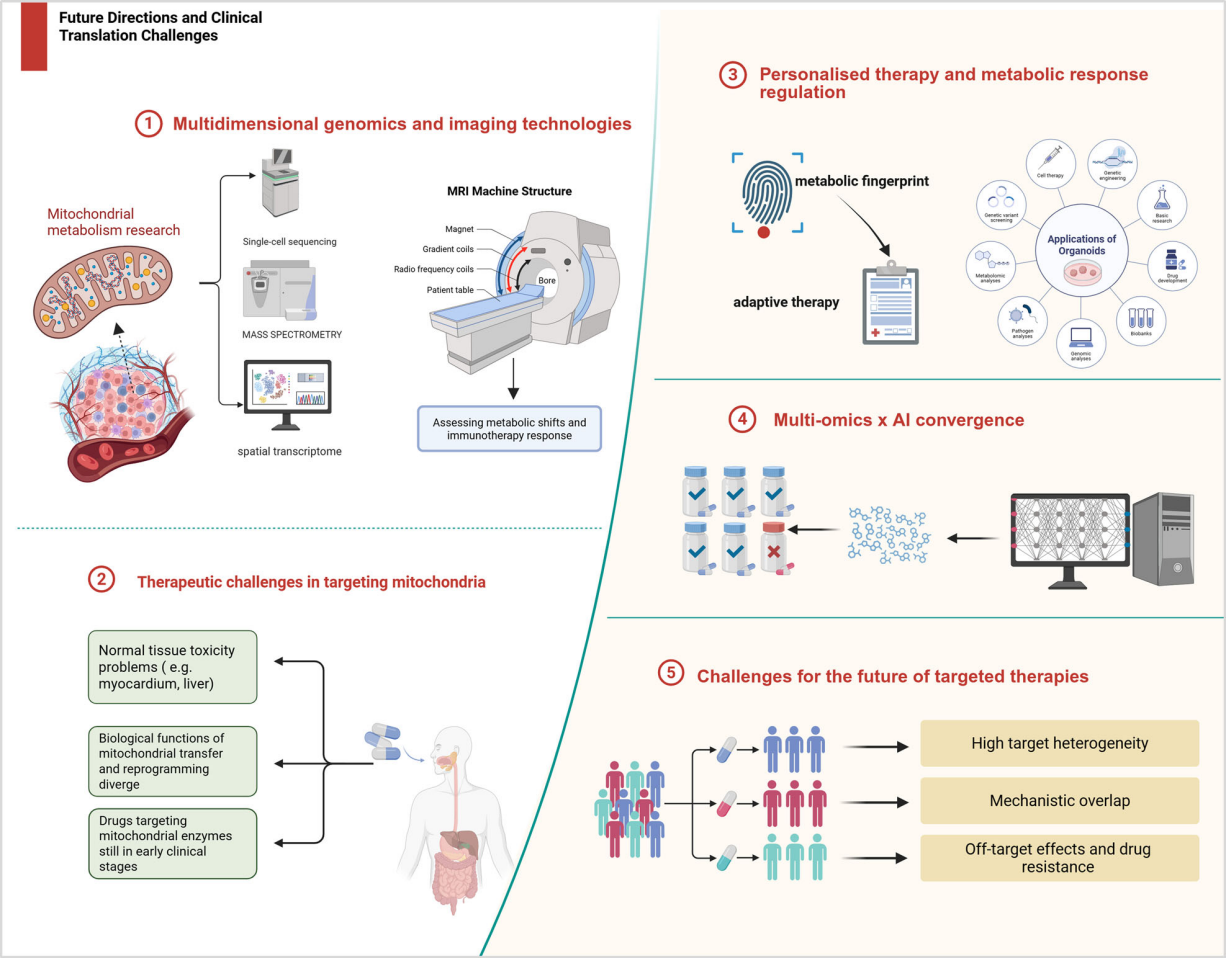
Future directions and clinical translation challenges. Future directions and clinical translation challenges in mitochondrial metabolism-targeted therapies. Key areas include: (1) Application of multidimensional genomics and imaging technologies to map mitochondrial metabolic shifts. (2) Therapeutic challenges such as normal tissue toxicity and biological complexity in targeting mitochondria. (3) Development of personalized therapies based on metabolic fingerprinting and organoid models. (4) Integration of multi-omics data with AI to refine therapeutic strategies. (5) Overcoming hurdles like target heterogeneity, mechanistic redundancy, and drug resistance in future targeted therapy development.

With a deeper understanding of mitochondrial metabolic reprogramming in CRC, both research and clinical fields are collectively advancing its translation from basic discoveries to precision therapies. However, this process still faces several technical bottlenecks, mechanistic controversies, and challenges related to therapeutic adaptability. This chapter systematically explores the current issues and future directions across four key dimensions: translational pathways, cutting-edge tools, personalized medicine, and emerging trends (Fig. 5).

### Multi-omics and imaging technologies: the trend towards integrated platforms in mitochondrial metabolism research

In recent years, the rapid advancement of multi-omics and functional imaging technologies has propelled mitochondrial metabolism research into a new phase of integration and system-level analysis [161–164]. As a foundational tool, metabolomics has been widely applied to identify characteristic metabolites in the blood of CRC patients, such as pyruvate, glutamate, and short-chain fatty acids [165]. Several of these metabolites have already been explored as candidate biomarkers and therapeutic targets in preclinical studies [165]. Meanwhile, single-cell RNA sequencing (scRNA-seq) has revealed that stem-like cell populations with elevated OXPHOS activity contribute to drug resistance and metastatic potential, providing direct evidence for the cellular basis of metabolic heterogeneity in CRC [166, 167].

Spatial omics technologies, particularly the 10x Visium platform, have enabled simultaneous profiling of mitochondrial metabolic states and immune cell infiltration patterns at anatomical resolution, facilitating the initial construction of spatially resolved metabolism–immune interaction maps [168].In parallel, proteomics is increasingly recognized as a critical complement to transcriptomic and metabolomic data. Proteogenomic studies have uncovered substantial differences in mitochondrial protein expression between primary and metastatic CRC, particularly in pathways related to energy metabolism, electron transport, and mitochondrial quality control mechanisms [169]. These investigations not only uncover dynamic changes in protein abundance and post-translational modifications but also help identify mitochondrial dysfunction signatures closely associated with therapeutic resistance.

Furthermore, the integration of proteomic, transcriptomic, and metabolomic data enables the reconstruction of multilayered regulatory networks that underlie mitochondrial and metabolic functions [170, 171]. This systems-level strategy facilitates the identification of biomarkers and therapeutic targets that may be missed by single-omics analyses, offering novel insights into functional heterogeneity and opportunities for precision interventions.

Meanwhile, emerging metabolic imaging techniques, such as hyperpolarized magnetic resonance imaging (HP-MRI) and photoacoustic imaging, can now track in real-time the pyruvate/lactate conversion ratio, mitochondrial membrane potential, and dynamic changes in ROS [172].

### Targeting mitochondria in therapy: toxicity, mechanistic ambiguities, and translational barriers

Although mitochondrial-targeted therapies have shown great potential in various cancers, they still face significant challenges in clinical advancement. The foremost issue is toxicity to normal tissues. Since mitochondrial metabolism is prevalent in normal high-energy-demand tissues, such as the heart and liver, OXPHOS inhibitors (e.g., IACS-010759) have shown dose-limiting toxicities (DLT) in some clinical trials, hindering their widespread application [173].

Furthermore, there remains controversy regarding the biological functions of mitochondrial transfer and reprogramming. Some studies suggest that introducing healthy mitochondria into cancer cells can enhance their sensitivity to ferroptosis inducers [174], while other studies indicate that mitochondrial fusion may activate protective metabolic axes (e.g., Nrf2), thereby promoting tumor growth [175].

Furthermore, therapeutic agents targeting mitochondrial enzymes such as SHMT2, PDH, and CPT1A remain largely in preclinical or early-phase clinical development, with limited data available on optimal delivery strategies, pharmacokinetics, and safety profiles [24]. Therefore, developing more selective and less toxic delivery systems, such as mitochondrial-targeted nanoparticles, will be a key direction for future advancements.

### Personalized treatment and metabolic response modulation: from organoids to real-time treatment adjustment

With the advancement of personalized medicine, CRC treatment strategies are gradually shifting from traditional molecular subtyping toward metabolic subtyping. Metabolic fingerprints (e.g., OCR/ECAR ratio, mtDNA copy number) are emerging as key indicators for assessing resistance risk [176, 177]alongside KRAS and BRAF mutation status.

Additionally, PDOs have been widely used for predicting drug sensitivity in CRC and can be combined with mitochondrial function dyes (e.g., JC-1, TMRM) to perform metabolic dependency-based individual screening [178]. The PDO-based metabolic drug screening platform is becoming an essential tool for the development of personalized strategies [179]. Studies have shown that colorectal cancer liver metastasis (CRLM) PDOs can highly preserve the histopathological and molecular characteristics of primary tumors while successfully capturing intra- and inter-patient heterogeneity [180]. In vitro drug sensitivity tests further confirmed that PDOs exhibit significant individual variability in response to monotherapy or combination chemotherapy, establishing a theoretical foundation for their application in personalized medicine [181].

However, despite their advantages in preserving tumor heterogeneity and genetic fidelity, PDOs face notable translational limitations. They lack critical components of the TME, including immune cells, fibroblasts, and vasculature [182]. Additionally, standard in vitro culture conditions fail to replicate hypoxia and nutrient deprivation, which are essential for shaping metabolic plasticity. These deficiencies may lead to underestimation of metabolic dependencies such as glutamine addiction or redox stress tolerance. Furthermore, PDOs are limited in capturing dynamic clonal evolution and adaptive resistance under prolonged therapeutic pressure [183].

To enhance translational relevance, recent innovations have integrated PDOs with autologous immune cell or stromal co-culture, and in vivo models such as PDO-derived xenografts (PDXs) and liver metastasis organoid models have been applied for validation. For example, the CeSta integrated model developed by Perron et al. successfully predicted cetuximab sensitivity in CRC PDX models by incorporating multi-omics data, providing a novel strategy for precision screening of targeted therapies [181].

Concurrently, real-time clinical adjustment strategies are emerging, combining organoid-informed predictions with longitudinal monitoring tools—such as tDNA for tracking metabolic mutation burden, and FDG-PET imaging to assess glucose metabolism [75].

In summary, PDOs offer a promising platform that bridges metabolic phenotyping and precision oncology. Nevertheless, their predictive power relies on the integration of multiple dimensions of biological information. These include immune cell dynamics, cellular metabolic activity, and the molecular evolution of tumor clones over time. Future directions should prioritize single-cell multi-omics, immune-tumor co-modeling, and real-time clinical feedback systems to unlock the full potential of organoid-informed metabolic therapy in CRC.

### Multi-omics × AI integration: driving the intelligent evolution of metabolic treatment strategies

AI and machine learning (ML) are becoming key drivers in the integration of multi-omics data. AI can extract metabolic dependency patterns from multi-dimensional omics (genomics, proteomics, metabolomics) to predict the response of mitochondrial metabolic subtypes to specific treatments [184, 185].

For example, the potential of AI in metabolic imaging and drug response prediction has been validated. One study proposed a drug response prediction method based on graph neural networks (GNN), which can integrate drug molecular graphs and gene expression data to predict the response of cancer cells to drugs and reveal their mechanisms of action [186].

In the future, integrating AI models with organoid platforms and ctDNA liquid biopsy systems is expected to form a closed-loop personalized metabolic treatment system, comprising “diagnosis → prediction → feedback → adjustment”.

### Future trends and controversies in metabolic targeting strategies

Despite the unprecedented promise of mitochondrial targeting and metabolic reprogramming therapies in CRC, several challenges remain. One major issue is high target heterogeneity. The metabolic pathways in CRC are not uniform, with different subtypes showing varying dependencies on OXPHOS, glycolysis, and lipid metabolism [26]. Precisely classifying these subtypes to identify the most effective therapeutic targets remains a significant challenge.

Another critical challenge is mechanistic overlap. Metabolic alterations in CRC are intricately connected with various signaling pathways, such as PI3K/Akt and Wnt [26]. As a result, targeting a single metabolic pathway may not lead to sustained therapeutic responses, as compensatory mechanisms can activate other pathways, undermining the treatment’s efficacy.

Finally, the issue of off-target effects and drug resistance complicates the development of mitochondrial-targeted therapies. Many OXPHOS inhibitors or SHMT2 inhibitors exhibit narrow therapeutic windows and significant side effects [187]. Moreover, successful data integration for translational applications is essential. It is necessary to establish multi-center, cross-platform data integration systems to strengthen the connection between basic research and clinical data, improving drug prediction accuracy and therapeutic consistency [188].

In the future, the focus of CRC treatment will gradually shift from a “driver gene-based” approach to a “metabolic ecosystem-based” approach. Building an interdisciplinary integration platform centered around mitochondrial function, incorporating technologies such as AI, organoids, imaging, and spatial omics, will be key to enhancing treatment precision and durability. In the face of controversies and challenges, it is essential to establish a more efficient “translational interface” between science, technology, and clinical practice, driving mitochondrial metabolic reprogramming therapy from a “potential pathway” to “clinical reality.”

## Conclusion

Mitochondrial metabolic reprogramming plays a central role in the therapeutic failure of CRC. Cancer cells modulate mitochondrial metabolism in response to treatment, activating alternative metabolic pathways and altering mitochondrial dynamics, thereby resisting the effects of drugs. Understanding these mechanisms is crucial for developing effective strategies to combat drug resistance. Targeting specific aspects of mitochondrial reprogramming, such as the activation of alternative metabolic pathways and changes in mitochondrial apoptosis mechanisms, may become key to enhancing the efficacy of existing therapies and developing new treatments.

To overcome therapeutic resistance, targeting metabolic pathways, combination therapies, and the development of drugs aimed at the tumor microenvironment offer new hope. With the advancement of mechanistic research and the progression of clinical trials, the treatment of CRC is moving toward more personalized and precise approaches, thereby providing patients with more effective and safer treatment options.

Despite these developments, significant obstacles remain, including tumor heterogeneity, complex resistance mechanisms, off-target effects, and limited predictive biomarkers. Future research should focus on the integration of multi-omics data, including proteomics, spatial transcriptomics, and metabolomics, in combination with AI-driven analyses. This integrated approach can help unravel the metabolic complexity of CRC and support the development of individualized treatment strategies. Among these omics tools, proteomic data provide important complementary insights into mitochondrial protein expression, enzyme activity, and post-translational modifications, helping to reveal functional aspects of metabolic plasticity and therapy resistance. Collaborative, interdisciplinary efforts are needed to translate these findings into clinical practice. Standardized diagnostic, therapeutic, and monitoring protocols based on mitochondrial metabolic phenotypes should be established to enhance treatment outcomes and patient quality of life.

Overall, this review provides a CRC-specific integration of mitochondrial metabolic reprogramming with mechanisms of therapy resistance. By highlighting mitochondria as both a mechanistic driver and actionable target, it offers a focused and translationally relevant perspective to guide future research and clinical strategies.

## Data Availability

No data was used for the research described in the article.
